# Coping with uncertainty in college: a TMIM-based study on incoming first-year university students

**DOI:** 10.3389/fpsyg.2026.1794392

**Published:** 2026-06-22

**Authors:** Jiaxin Lin, Manyu Liao, Zhuowen Feng, Haiyang Gao

**Affiliations:** 1 College of Literature and News Communication, Guangdong Ocean University, Zhanjiang, Guangdong, China; 2 Faculty of Humanities and Social Sciences, City University of Macau, Taipa, Macao SAR, China; 3 Faculty of Chemistry and Environmental Science, Guangdong Ocean University, Zhanjiang, Guangdong, China

**Keywords:** incoming first-year university students, information overload, information seeking, new media literacy, theory of motivated information management (TMIM)

## Abstract

Guided by the Theory of Motivated Information Management (TMIM), this study examines how Chinese incoming first-year university students (*N* = 417) manage pre-enrollment uncertainty through information seeking. Data were analyzed using structural equation modeling. Results show that uncertainty discrepancy positively predicts anxiety. Anxiety was positively associated with outcome expectancy but negatively associated with efficacy expectancy. Both cognitive appraisals significantly predict information-seeking behavior. Furthermore, information overload serves as a moderator that strengthens the relationship between anxiety and outcome expectancy. Finally, consumption-oriented new media literacy exerts differentiated moderating effects: it enhances the impact of efficacy expectancy on information seeking while reducing the influence of outcome expectancy. By integrating information overload and consumption-oriented new media literacy into the TMIM framework, this study reveals how incoming first-year university students manage uncertainty in complex information environments.

## 1 Introduction

The scale of higher education in China expanded rapidly. According to the Statistical Bulletin of National Education Development (2024),^1^ the number of students enrolled in higher education has exceeded 48 million, and the gross enrollment ratio exceeded 60%. Each year, more than 11 million students enter undergraduate and graduate programs. Incoming first-year university students represent a critical transitional group between secondary and higher education. During this transition, they face challenges related to environmental adaptation, role adjustment, and future developmental choices. As a result, their identity formation and behavioral patterns are often accompanied by high levels of uncertainty and increased information needs (Yang, 2017). At the same time, the Internet has become a central platform through which university students obtain information and engage in social interaction (Gong et al., 2025). According to the Statistical Report on the Development of the Internet in China,^2^ individuals aged 20 to 29 account for 12.8% of all Internet users, with an average daily online time of approximately 4.4 hours. Survey data from nearly 40,000 university students further indicate that online media are deeply embedded in students' academic and everyday lives (Wang, 2022). In this context, information serves as a critical resource for responding to environmental change and reducing cognitive ambiguity. It plays an essential role in incoming first-year university students' adjustment to university life.

With the growing reliance on digital platforms for information dissemination in university settings (Xu et al., 2023), incoming first-year university students often encounter information gaps related to academic structures, access to campus resources, and future career or developmental pathways. These gaps may negatively affect students' adjustment experiences and increase psychological anxiety (Xing et al., 2021). Incoming first-year university students from different backgrounds also differ in their information source preferences and emotional responses. For example, first-generation college students tend to rely more on formal sources of support, such as librarians and counseling centers. In contrast, continuing-generation students are more likely to seek information from peers and friends (Brinkman and Smith, 2021). In addition, pre-enrollment orientation or bridging programs designed to support the transition into higher education may require students to make additional information-related decisions. These demands can increase experiences of information overload and anxiety.

The Theory of Motivated Information Management (TMIM) offers a systematic framework for explaining how individuals manage information under conditions of uncertainty. The theory describes a three-phase process, including the interpretation phase, the evaluation phase, and the decision phase. TMIM has been widely applied and validated in health, family, and organizational contexts (Afifi and Weiner, 2004; Kuang and Wilson, 2021). However, relatively few studies have examined its application to information-seeking behavior during the transition from secondary school to university. This study focuses on information seeking during the pre-enrollment transition period rather than post-enrollment adaptation behavior.

Unlike many health-related uncertainty contexts, the pre-enrollment transition to university involves a set of concrete, time-sensitive, and preparatory tasks. Incoming students need to obtain information about academic arrangements, campus resources, accommodation, peer networks, and future developmental opportunities. Such uncertainty is therefore not only threatening but also actionable. Information seeking may be perceived as a practical strategy for preparing for university life and regaining a sense of control.

Moreover, in highly mediated information environments, contextual factors such as consumption-oriented new media literacy and information overload may shape how incoming first-year university students perceive and respond to uncertainty. New Media Literacy reflects individuals' ability to interpret, evaluate, and integrate complex information, whereas information overload refers to the extent to which excessive information disrupts cognitive processing and decision-making (Shen et al., 2023; Whelan et al., 2020). Accordingly, this study extends TMIM to the context of incoming first-year university students and clarifies how they manage uncertainty in complex information environments. Grounded in the TMIM framework, the study examines the model's applicability in a specific educational and digital context. It further introduces consumption-oriented NML, specifically functional and critical consumption, and information overload as moderating variables to examine whether and how these factors shape the information-management process. Through this approach, the study identifies distinctive patterns of information seeking among incoming first-year university students.

## 2 Theoretical framework

### 2.1 Theory of motivated information management

**Figure 1:**
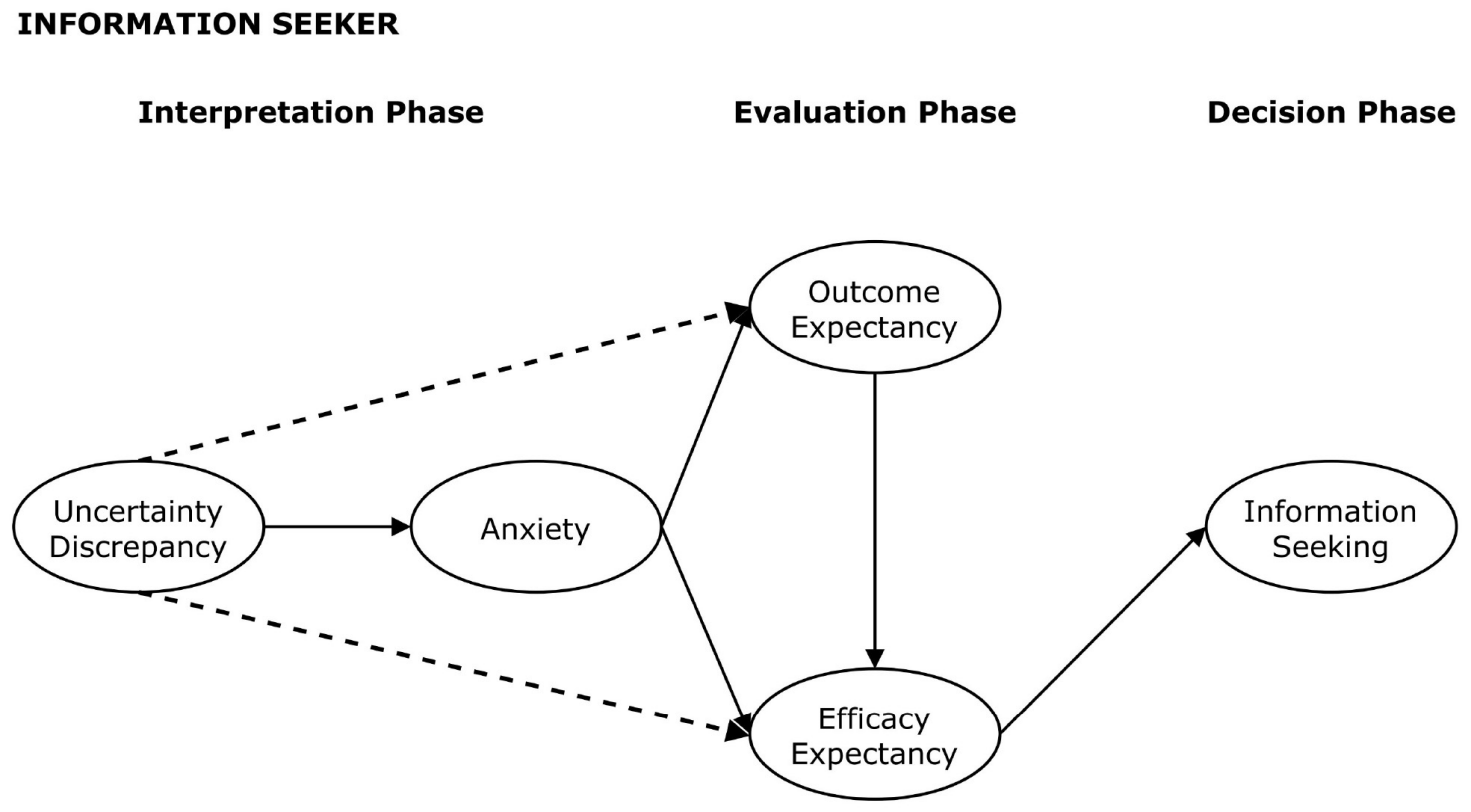
The original theoretical model of TMIM.

TMIM, proposed by Afifi and Weiner (2004), explains how individuals manage information under conditions of uncertainty. The theory outlines a three-phase process: interpretation, evaluation, and decision. These phases are driven by five core constructs: uncertainty discrepancy, anxiety, outcome expectancy, efficacy expectancy, and information management behavior (Fowler and Afifi, 2011). The relationships among these constructs are illustrated in Figure 1.

TMIM posits that information behavior is shaped jointly by cognitive evaluations and emotional responses. The process begins when individuals perceive a gap between what they know and what they wish to know. This perceived gap, referred to as uncertainty discrepancy, generates anxiety and activates an evaluative process. During the evaluation phase, individuals assess both the expected outcomes of seeking information and their ability to successfully obtain and process that information. Based on these assessments, they select an information management strategy, such as information seeking, information avoidance, or selective engagement with specific information sources.

TMIM has been widely applied across diverse contexts, including health communication, education, career decision-making, and social relationships (Afifi and Weiner, 2004; Kuang and Wilson, 2021). For example, Li et al. (2020) examined parental information management in childhood vaccination and found that uncertainty discrepancy, outcome expectancy, and efficacy expectancy significantly influenced both information seeking and information avoidance. Similarly, Feng et al. (2025) demonstrated that these core TMIM variables predicted Chinese university students' information behaviors regarding graduation-related decisions. Kim et al. (2022) further applied TMIM to interpersonal contexts by analyzing uncertainty management among students with social anxiety disorders.

These findings indicate that TMIM is well suited for examining the transition to university, a period marked by pervasive uncertainty. Incoming first-year university students commonly encounter ambiguous academic expectations, unfamiliar social contexts, and uncertain future trajectories. As such, this transitional context provides a suitable setting for applying TMIM to examine how students manage uncertainty through information-related behaviors.

#### 2.1.1 Interpretation phase in TMIM

Within TMIM, the interpretation phase marks the point at which individuals recognize a discrepancy between their current and desired knowledge states. This discrepancy, known as uncertainty discrepancy, is the central mechanism of this phase (Afifi and Weiner, 2004). When the perceived gap exceeds an individual's tolerance threshold, it disrupts cognitive balance and triggers negative emotional responses, particularly anxiety. In this study, uncertainty refers specifically to the academic, daily-life, and future-related uncertainties that incoming first-year university students experience prior to enrollment. Empirical research consistently shows a positive association between uncertainty discrepancy and anxiety. For instance, Rauscher and Hesse (2014) found that greater uncertainty regarding family health history was associated with higher levels of anxiety. Similar patterns have been observed in studies of adolescents' sexual health information seeking, where larger information gaps predicted stronger anxiety responses (Jayasundara, 2021).

For incoming first-year university students, gaps in access to social and institutional support resources are common during the transition to university. From a TMIM perspective, such limitations may exacerbate perceived uncertainty discrepancies. Prior research has demonstrated that insufficient social and emotional support is associated with heightened separation anxiety and adjustment difficulties among new students (Claborn and Kane, 2019). These challenges reflect not only difficulties in adjusting to the institutional environment but also unmet needs related to the establishment of effective social support networks. Such adjustment deficits have been shown to undermine students' psychological well-being and increase the risk of attrition during the first year of college (Claborn and Kane, 2019).

Research focusing on specific student groups further supports this relationship. Brinkman and Smith (2021) found that first-generation college students experience higher anxiety during information seeking due to a lack of familial higher education experience. Their reliance on formal institutional sources highlights the psychological burden created by uncertainty discrepancy. In addition, Xiong et al. (2024) demonstrated that intolerance of uncertainty is a significant antecedent of anxiety among university students.

#### 2.1.2 Evaluation phase in TMIM

The evaluation phase represents the core cognitive component of TMIM. In this phase, individuals assess potential information management behaviors through two key variables: outcome expectancy and efficacy expectancy (Afifi and Weiner, 2004). Under conditions of anxiety, individuals evaluate both the anticipated outcomes of seeking information and their perceived ability to perform this behavior. Outcome expectancy refers to individuals' evaluations of the potential benefits or costs of information-management actions. In contrast, efficacy expectancy reflects individuals' confidence in their ability to successfully obtain and process information. It includes three dimensions: communication efficacy, coping efficacy, and target efficacy.

Early empirical studies found that anxiety negatively predicted outcome expectancy. For example, research on eldercare communication showed that anxiety reduced positive outcome expectations (Fowler and Afifi, 2011). Similar negative relationships were reported in studies of family health communication (Rauscher and Hesse, 2014), vaccination-related information conflict (Li et al., 2020), and social anxiety contexts (Kim et al., 2022). These findings were especially robust in health-related contexts. However, subsequent research suggests that this relationship may be context-dependent. During the COVID-19 pandemic, Ju et al. (2022) found that anxiety positively predicted outcome expectancy. In that context, information seeking was perceived as a necessary tool for risk reduction. Anxiety, therefore, increased the perceived value of seeking information rather than discouraging it.

The university transition shares several characteristics with other uncertainty-rich transitional contexts. Both involve major life changes associated with unfamiliar environments, elevated uncertainty, and increased demands for adaptation (Ramírez-Martínez et al., 2024). In addition, these contexts are often characterized by information overload and uncertainty regarding information quality. Incoming first-year university students are frequently exposed to large amounts of information from official channels, peers, and social media platforms, where variations in accuracy, consistency, and timeliness may occur (Wang and Chang, 2023; Zeng et al., 2024). Under such conditions, information seeking may be viewed as an important strategy for reducing uncertainty and facilitating adaptation.

Recent research further suggests that anxiety may increase the perceived value of information acquisition during periods of major environmental change and uncertainty. Studies have shown that anxious individuals are more likely to engage with information when doing so is perceived as helpful for adapting to changing environments and preparing for future challenges (Charpentier et al., 2022). This interpretation is also consistent with proactive coping and anticipatory socialization perspectives, which suggest that individuals actively seek information before entering unfamiliar roles or environments in order to facilitate adaptation and reduce uncertainty (Fetherston, 2017). Similarly, during the transition into university, students may perceive information seeking as a proactive coping strategy that helps them prepare for upcoming academic, social, and institutional demands. Therefore, anxiety may positively enhance outcome expectancy in this context.

By contrast, anxiety shows a consistently negative relationship with efficacy expectancy. Afifi and Afifi (2009) found that adolescents' anxiety about communicating with parents significantly reduced communication and target efficacy. Meta-analytic evidence further confirms that anxiety undermines all three dimensions of efficacy expectancy (Kuang and Wilson, 2021). This pattern also appears among incoming first-year university students. Brinkman and Smith (2021) reported that anxiety about navigating university systems weakened students' confidence in acquiring information. Similar findings have been reported in studies of matriculation anxiety (Zhang and Cui, 2021) and online information retrieval anxiety (Eldakar and Kenawy, 2020; Yari Zanganeh and Hariri, 2018).

TMIM also indicates a positive relationship between outcome expectancy and efficacy expectancy. When individuals perceive information seeking as valuable, they are more likely to believe they can perform it effectively (Afifi and Weiner, 2004). Empirical studies support this relationship. Kuang and Gettings (2023) found that positive outcome expectations strengthened efficacy beliefs in aging-related information management. Gettings and Kuang (2021) reported similar effects in communication contexts. Among incoming first-year university students, perceiving academic information seeking as beneficial is therefore expected to enhance confidence in information processing abilities.

#### 2.1.3 Decision phase in TMIM

The decision phase represents the behavioral enactment stage of TMIM. At this stage, individuals select specific information management strategies, such as information seeking or information avoidance. Anxiety influences these choices indirectly through outcome expectancy and efficacy expectancy, while both cognitive evaluations also exert direct effects on behavior (Afifi and Weiner, 2004).

Among these pathways, efficacy expectancy plays a central role in shaping information behavior, as it determines whether individuals perceive themselves as capable of managing the information seeking process (Afifi and Weiner, 2004). Individuals who believe they can successfully acquire and process information are more likely to engage in proactive information seeking, whereas low efficacy beliefs increase the likelihood of avoidance. Empirical studies consistently support this relationship across health, digital, and educational contexts (Kim et al., 2022; Afifi and Weiner, 2006). Research focusing on university students similarly shows that higher efficacy beliefs are associated with more proactive information seeking strategies (Feng et al., 2025).

Outcome expectancy also plays a direct role in shaping information management decisions. TMIM suggests that positive outcome evaluations promote information seeking (Afifi and Weiner, 2004), a relationship that has been empirically supported across diverse contexts, including family communication (Afifi and Afifi, 2009), genetic health information seeking (Yoon and Theiss, 2022), and financial decision-making (Fowler et al., 2018). Extending this logic to the educational transition context, anticipating practical benefits from seeking academic information is expected to increase incoming first-year university students' engagement in proactive information-seeking behavior, such as consulting instructors or using digital platforms.

#### 2.1.4 The moderating role of information overload

Information overload refers to a situation where the amount, quality, or time demands of the information encountered exceed an individual's cognitive processing capacity. This leads to difficulties in filtering information, decreased decision-making efficiency, and increased cognitive load (Eppler and Mengis, 2004). Cognitive load theory provides core theoretical support for this concept (Sweller, 1988). According to the theory, human cognitive resources are limited, and excessive information occupies this finite cognitive space, resulting in reduced processing efficiency. Recent research during the pandemic has further expanded the understanding of this concept (Laato et al., 2020).

The inclusion of information overload in this study is supported by both practical and theoretical reasons. First, incoming first-year university students, during the pre-enrollment transition, must deal with an influx of information from multiple sources, including academics, daily life, and social interactions. This makes them highly vulnerable to information overload. Whelan et al. (2020) confirmed that social media information overload negatively impacts students' learning-related information behaviors. Since information seeking is a key behavior for students adapting to campus life, it is likely affected by information overload as well. Second, in the TMIM framework, anxiety is a key antecedent that influences both outcome expectancy and efficacy expectancy (Afifi and Weiner, 2004). Information overload, as an external cognitive stressor, may alter the mechanism by which anxiety influences the evaluation phase (Lee et al., 2016). Conceptually, information overload may influence the two appraisal pathways in different ways. It may be especially relevant to outcome expectancy because excessive, inconsistent, or fragmented information can heighten students' awareness of ambiguity and increase the perceived value of further clarification. At the same time, its role in shaping efficacy expectancy may be less straightforward because efficacy judgments are also grounded in students' prior information skills, communication confidence, coping resources, and access to supportive others. Therefore, information overload has the potential to moderate the relationships between anxiety and both outcome expectancy and efficacy expectancy.

#### 2.1.5 The moderating role of consumption-oriented new media literacy

New media literacy refers to an individual's ability to identify, access, critique, produce, and share information in new media environments (Shen et al., 2023). Its meaning has evolved from a focus on information reception, analysis, and critique in traditional media to a more multidimensional structure that equally values both consumption and production (Livingstone, 2004). This structure includes four key dimensions: functional consumption, critical consumption, functional prosuming, and critical prosuming (Chen et al., 2011; Lin et al., 2013). In this study, we distinguish between consumption-oriented NML and prosuming-related NML to clarify the specific competencies under investigation. Consumption-oriented NML refers specifically to abilities related to functional consumption, such as accessing and identifying information, and critical consumption, such as evaluating and judging information. In contrast, prosuming-related NML involves functional prosuming and critical prosuming, which focus on content creation and sharing.

Research indicates that different aspects of new media literacy, particularly functional prosuming and critical consumption, play a crucial role in enabling individuals to critically evaluate information and engage in participatory behaviors (Shen et al., 2023). On the other hand, a lack of these skills can lead to confusion and reduced effectiveness in information processing (Chen and Huang, 2019). Given that incoming first-year university students in the pre-enrollment stage are primarily information seekers rather than content producers, this study focuses specifically on consumption-oriented new media literacy as the relevant competency for information acquisition and evaluation.

Integrating consumption-oriented new media literacy into the TMIM framework has theoretical value, as it may serve as a key variable moderating the transition from the evaluation phase to the decision phase (Lee et al., 2015). According to TMIM, efficacy expectancy and outcome expectancy are critical drivers that move individuals into the decision phase to engage in information seeking (Afifi and Weiner, 2004). However, this transition from motivation to action is not automatic and depends critically on individuals' competencies. New media literacy involves competencies in information retrieval, critical evaluation, integration, and expression, which are fundamental to effective information management in digital environments (Chen et al., 2018). Theoretically, the level of these competencies determines whether individuals can effectively translate positive assessments (such as high efficacy expectancy and positive outcome expectancy) into actual information seeking. In this sense, new media literacy is likely to play a moderating role between the evaluation and decision phases, as variation in these competencies may condition the extent to which evaluative judgments are translated into information seeking. In particular, even when individuals hold positive evaluations, differences in new media literacy may shape how effectively these motivations are enacted in digital contexts characterized by high informational complexity and cognitive demands (Chen and Huang, 2019).

Based on this reasoning, the present study proposes that consumption-oriented new media literacy, hereafter referred to as C-NML, moderates the relationships between the core variables in the evaluation phase and information-seeking behavior in the decision phase among incoming first-year university students.

### 2.2 Conceptual framework and hypotheses

Based on the above analysis, this study proposes the following research hypotheses:

H1: Uncertainty discrepancy is positively associated with anxiety.

H2: Anxiety is positively associated with outcome expectancy.

H3: Anxiety is negatively associated with efficacy expectancy.

H4: Outcome expectancy is positively associated with efficacy expectancy.

H5: Efficacy expectancy is positively associated with information seeking.

H6: Outcome expectancy is positively associated with information seeking.

H7a: Information overload moderates the relationship between anxiety and outcome expectancy.

H7b: Information overload moderates the relationship between anxiety and efficacy expectancy.

H8a: Consumption-oriented NML moderates the relationship between efficacy expectancy and information seeking.

H8b: Consumption-oriented new media literacy moderates the relationship between outcome expectancy and information seeking.

**Figure 2:**
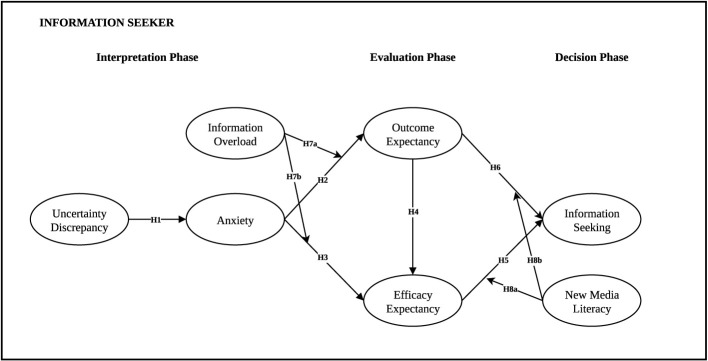
The proposed research model based on TMIM in this paper.

In summary, this study, grounded in the three-phase framework of TMIM and set in the context of university transition, introduces two key moderating variables: information overload and consumption-oriented new media literacy. These theoretical foundations lead to an integrated model with eight research hypotheses, as shown in Figure 2. The model outlines the primary pathway, where uncertainty discrepancy is associated with anxiety, which then, through the dual cognitive evaluations of outcome expectancy and efficacy expectancy, drives information seeking. The model also includes hypotheses about moderating effects: information overload moderates the relationship between the interpretation and evaluation phases, while consumption-oriented new media literacy moderates the relationship between the evaluation phase and the decision phase. The next section presents the empirical tests based on this model.

## 3 Materials and methods

### 3.1 Data collection and sampling

The study protocol was reviewed and approved by the Academic Committee of Guangdong Ocean University. Participation was voluntary, and informed consent was obtained from all respondents prior to survey completion. Participants were only allowed to proceed to the questionnaire after reviewing the informed consent statement and indicating their agreement electronically.

This study targeted incoming first-year university students. A snowball sampling method was employed, and the survey was administered via the Questionnaire Star platform. The survey link was distributed through social media platforms, including WeChat groups for new students, WeChat Moments, Douban, Xiaohongshu, and other platforms. Data collection took place from June 6 to August 31, 2025, spanning the period from shortly after the conclusion of the national college entrance examination to the weeks immediately preceding university enrollment.

All 501 returned questionnaires underwent a quality-control screening process. Responses with a completion time of less than 90 seconds were excluded, and cases that failed the attention-check questions were subsequently removed. After these procedures, 417 valid questionnaires were retained, yielding a valid questionnaire rate of 83.2%. The final sample comprised participants from 28 provincial-level administrative regions in China. The distribution of demographic characteristics is presented in Table 1.

**Table 1:** Demographic variables of participants.

Demographic characteristics	Category	Count (n)	Percentage (%)
Gender	Female	269	64.51%
	Male	148	35.49%
Education	Diploma	27	6.47%
	Bachelor's	320	76.74%
	Master's	69	16.55%
	Doctoral	1	0.24%
Channels for university information (multiple choice questions)	Douyin	294	70.5%
	Xiaohongshu	326	78.18%
	WeChat	320	76.74%
	AI Tools (Doubao, Tencent Yuanbao, DeepSeek, ChatGPT, Kimi, etc.)	124	29.74%
	Baidu Tieba	98	23.5%
	University Official Websites	271	64.99%
	Zhihu	58	13.91%
	Weibo	84	20.14%
	Douban	35	8.39%
	Others (Bilibili, QQ Groups, Peers, Senior Students)	15	3.6%

### 3.2 Measures

The core variables in this study were measured using well-established scales widely used in prior research. A standard cross-cultural adaptation procedure was followed to ensure the suitability of the instruments for the Chinese context. First, the original English scales were translated from English into Chinese and then back-translated into English. Any discrepancies in meaning between the original and back-translated versions were resolved through discussion and comparison, resulting in a preliminary Chinese version of the questionnaire.

To further improve the clarity and cultural appropriateness of the items, two pilot tests were conducted among incoming first-year university students. The first pilot study (*N* = 40) focused on evaluating item wording and comprehensibility, and revisions were made based on participant feedback. A second pilot study (N = 50) was subsequently conducted to assess item performance and internal consistency. Based on the results of item analysis and reliability checks, several items were further refined. The final scales showed satisfactory semantic clarity and contextual appropriateness for use among Chinese first-year university students.

In addition, several procedural remedies were adopted to reduce the risk of common method bias (Podsakoff et al., 2003). The survey was conducted anonymously and voluntarily, and participants were informed that there were no right or wrong answers. Established scales and pilot-tested items were used to improve clarity and reduce ambiguity. Furthermore, items measuring different constructs were separated in the questionnaire to reduce respondents' ability to infer the hypothesized relationships among variables. Attention-check items and response-time screening were also employed to improve overall data quality.

#### 3.2.1 Uncertainty discrepancy

Uncertainty discrepancy was measured using a scale adapted from Afifi and Weiner (2006), employing a 7-point Likert scale (1 = strongly disagree, 7 = strongly agree). The scale included three items (M = 5.738, SD = 0.784, α = 0.729). Sample items are: “I know less about the future university than I expected,” “I wish I had more relevant information about the future university,” and “I wish I had a better understanding of the university I will attend.”

#### 3.2.2 Anxiety

Anxiety was measured using a scale adapted from Afifi and Weiner (2006), also with a 7-point Likert scale (1 = strongly disagree, 7 = strongly agree). This dimension included three items (M = 5.101, SD = 1.217, α = 0.916). Sample items are: “I feel anxious when I think about how little I know about the university”, “I feel restless when faced with school information I have not yet understood”, and “Having too little information about the university makes me anxious”.

#### 3.2.3 Outcome expectancy

Outcome expectancy was measured using a scale adapted from Li et al. (2025), with a 5-point Likert scale (1 = strongly disagree, 5 = strongly agree). This dimension consisted of three items (M = 4.052, SD = 0.565, α = 0.721). Sample items include: “I believe actively searching for information about the university will lead to positive outcomes”, “I believe searching for university-related content can give me a clearer picture of university life, and this searching behavior is beneficial”, and “I believe searching for university-related content can directly answer my questions about various aspects of the university”.

#### 3.2.4 Efficacy expectancy

Efficacy expectancy was also measured using a scale adapted from Li et al. (2025) with a 5-point Likert scale (1 = strongly disagree, 5 = strongly agree). The scale included two sub-dimensions: communication efficacy and coping efficacy, each with three items, totaling six items (M = 3.651, SD = 0.673, α = 0.848). Sample items for communication efficacy include: “I can proactively communicate with others and efficiently obtain information about the university”, “I can ask questions clearly and accurately to others, thus easily obtaining the university information I want”, and “I make good use of asking targeted questions to others to get university information more quickly”. Sample items for coping efficacy are: “Even if the university information I find isn't ideal, I can quickly adjust my emotions and view it rationally”, “I can handle any university information I find effectively”, and “When faced with university information, I am always able to handle it calmly and prepare accordingly”.

#### 3.2.5 Information seeking

Information seeking was measured using a scale adapted from Hovick et al. (2014) with a 7-point Likert scale (1 = strongly disagree, 7 = strongly agree). This dimension contained four items (M = 5.479, SD = 0.992, α = 0.896). A sample item is: “Recently, I have actively searched for university information to prepare for enrollment”.

#### 3.2.6 Information overload

Information overload was assessed using a scale adapted from Laato et al. (2020) with a 5-point Likert scale (1 = strongly disagree, 5 = strongly agree). This dimension included three items (M = 3.194, SD = 1.003, α = 0.887). Sample items include: “An excessive amount of information about the university I will attend on new media actually makes me feel more unfamiliar with the university” and “I am exposed to too much university information from new media, which is difficult to digest and makes my understanding of the university more vague”.

#### 3.2.7 Consumption-oriented new media literacy

The consumption-oriented new media literacy scale was adapted from the new media literacy scale developed by Shen et al. (2023), with items measured on a five-point Likert scale. The original scale was constructed based on the four-dimensional new media literacy framework proposed in previous research (Lin et al., 2013; Chen et al., 2018). The original instrument consisted of 20 items covering functional consumption, critical consumption, functional prosuming, and critical prosuming dimensions.

Given the specific focus of the present study on incoming first-year university students' information-seeking behavior prior to university enrollment, the scale was contextually adapted by retaining only the 10 items corresponding to the functional consumption and critical consumption dimensions. This adaptation was theoretically guided by the present study's focus on the transition from the evaluation phase to the decision phase in TMIM, which primarily concerns individuals' information acquisition, comprehension, evaluation, and uncertainty reduction rather than media production or participatory content creation. Previous new media literacy research has distinguished consumption-oriented literacy from prosuming-related literacy and conceptualized them as related but distinct dimensions with different behavioral emphases (Lin et al., 2013; Chen et al., 2018). Specifically, functional and critical consumption literacy primarily reflect individuals' abilities to access, understand, analyze, evaluate, and integrate media information, whereas prosuming-related dimensions emphasize content production, distribution, participation, and media creation.

Methodological research has further suggested that contextualized scale adaptation is appropriate when established instruments are applied to specific populations, behavioral outcomes, and situational contexts (Ambuehl and Inauen, 2022). In the present study, incoming first-year university students during the pre-enrollment stage primarily engaged in searching for, interpreting, and evaluating university-related information rather than generating or disseminating media content. Therefore, retaining only the consumption-oriented dimensions improved the contextual relevance of the measurement and reduced potential conceptual overlap between media production behaviors and the focal information-seeking outcome. The retained items were selected based on their direct relevance to information access, critical evaluation, and information-processing competencies in digital environments.

To further evaluate the appropriateness of the adapted scale, a confirmatory factor analysis (CFA) was conducted to assess its factor structure. The results indicated that the adapted measurement model showed an acceptable overall fit. Responses were recorded on a five-point Likert scale (1 = strongly disagree, 5 = strongly agree). The adapted scale demonstrated acceptable internal consistency (M = 4.000, SD = 0.457, α = 0.787). Sample items include: “I am proficient in using functions such as keywords and filters to efficiently search for university information” and “When using new media to acquire university information, I pay attention to whether the information source is credible and legitimate”.

## 4 Results

Data analysis was conducted using SPSS 27.0 and AMOS 26.0. First, SPSS 27.0 was used to perform descriptive statistics (mean and standard deviation calculations), reliability tests (Cronbach's α), and moderation analyses using Hayes's PROCESS macro (Model 1). Second, AMOS 26.0 was used to estimate a structural equation model (SEM) to test the main effect pathways of TMIM. Model fit was assessed using the following fit indices: χ^2^/*df*, CFI, and RMSEA. The bias-corrected bootstrap method (with 5,000 resamples) was applied to calculate the 95% confidence intervals for the indirect effects. All continuous variables involved in the interaction terms were mean-centered before the moderation analyses.

### 4.1 Assessment of measurement model reliability, validity, and factor loadings

Before testing the SEM pathways, the suitability of the data for factor analysis was assessed using the Kaiser-Meyer-Olkin (KMO) measure and Bartlett's test of sphericity. The results showed a KMO value of 0.855, exceeding the recommended threshold of 0.80. Bartlett's test of sphericity was significant (χ^2^ = 6301.665, df = 496, *p* < 0.001), indicating that the data were appropriate for factor analysis. To assess the potential influence of common method bias, Harman's single-factor test was conducted (Podsakoff et al., 2003). All measurement items were entered into an unrotated exploratory factor analysis. The results showed that the first factor accounted for 21.919% of the total variance, which was well below the recommended threshold of 40%, suggesting that common method bias was unlikely to be a serious concern in the present study.

The reliability and validity of the primary latent constructs included in the SEM model were then assessed. The results of these measurement assessments are presented in Table 2. Convergent validity and composite reliability were evaluated following established criteria in the SEM literature. Specifically, composite reliability (CR) values above 0.70 are generally considered indicative of adequate internal consistency (Hair, 2009). In the present study, the CR values for all latent variables exceeded this threshold. In particular, anxiety (CR = 0.916), information seeking (CR = 0.897), and efficacy expectancy (CR = 0.848) all demonstrated CR values above 0.80, indicating good internal consistency for these constructs (Fornell and Larcker, 1981). Although the AVE values for outcome expectancy (AVE = 0.473) and efficacy expectancy (AVE = 0.482) were slightly below the recommended cutoff of 0.50, they were close to the threshold. Given that their composite reliability values were satisfactory, the convergent validity of these constructs was considered marginally acceptable, consistent with the criteria proposed by Fornell and Larcker (1981). With respect to item loadings, the standardized factor loadings ranged from 0.531 to 0.899, exceeding the minimum acceptable level of 0.50 (Hair, 2009). In addition, all critical ratios exceeded 1.96 (*p* < 0.001), indicating that each measurement item significantly represented its intended latent construct (Kline, 2023). Overall, the reliability and validity indices of the primary SEM measurement model were generally acceptable and provided an adequate basis for subsequent structural model analysis.

**Table 2:** Assessment results of the measurement model: reliability, validity, and factor loadings.

Item	Standardized factor loadings	Cronbach's α	CR	AVE
UD1	0.531	0.729	0.747	0.504
UD2	0.732			
UD3	0.834			
AN1	0.867	0.916	0.916	0.785
AN2	0.891			
AN3	0.899			
OE1	0.714	0.721	0.728	0.473
OE2	0.695			
OE3	0.651			
EE1	0.692	0.848	0.848	0.482
EE2	0.738			
EE3	0.691			
EE4	0.669			
EE5	0.704			
EE6	0.670			
IS1	0.845	0.896	0.897	0.686
IS2	0.855			
IS3	0.850			
IS4	0.759			

The critical ratio (C.R.) for all standardized factor loadings exceeded the threshold of 1.96, *p* < 0.001. UD, Uncertainty Discrepancy; AN, Anxiety; OE, Outcome Expectancy; EE, Efficacy Expectancy; IS, Information Seeking.

Subsequently, the discriminant validity of the measurement model was assessed by comparing the square roots of the AVE with the correlation coefficients among the latent variables (see Table 3). As shown in the table, the square root of the AVE for each latent variable (bolded values on the diagonal) was greater than its correlations with other latent variables. For example, the square root of the AVE for uncertainty discrepancy (0.710) exceeded its correlations with anxiety (0.269), outcome expectancy (0.049), and other variables. Similarly, the square root of the AVE for anxiety (0.886) was greater than its correlations with all other variables (the highest being 0.159).

**Table 3:** Correlation coefficients and discriminant validity.

Latent variable (LV)	UD	AN	OE	EE	IS
UD	**0.710**				
AN	0.269^**^	**0.886**			
OE	0.049	0.159^**^	**0.688**		
EE	-0.005	-0.015	0.230^**^	**0.694**	
IS	0.005	0.014	0.240^**^	0.412^**^	**0.828**

1. The boldfaced values on the diagonal are the square roots of the average variance extracted (AVE) for each latent variable (calculated from the AVE values in Table 2); the off-diagonal values are the Pearson correlation coefficients between the latent variables.

2. ^**^Denotes *p* < 0.01.

3. UD, Uncertainty Discrepancy; AN, Anxiety; OE, Outcome Expectancy; EE, Efficacy Expectancy; IS, Information Seeking.

Notably, the AVE values for outcome expectancy (0.473) and efficacy expectancy (0.482) were slightly below the conventional threshold of 0.50. This suggests that the convergent validity of these two constructs was marginal and should be interpreted with caution. Nevertheless, their composite reliability values exceeded 0.70, and all standardized factor loadings were above 0.50, indicating that the measurement quality of these constructs remained acceptable for subsequent analysis.

In addition, several bivariate correlations among the latent variables were relatively modest. However, this pattern does not necessarily contradict the TMIM framework. TMIM conceptualizes information management as a staged and mediated process rather than as a set of uniformly strong direct associations. In particular, uncertainty discrepancy is expected to influence outcome expectancy mainly through anxiety and subsequent cognitive appraisal, rather than through a strong direct association. Therefore, the modest correlation between uncertainty discrepancy and outcome expectancy may reflect the distal and indirect nature of this pathway. Nevertheless, the relatively weak associations among some distal constructs suggest that these theoretical links should be interpreted with appropriate caution.

These results suggest that the five latent variables demonstrated acceptable discriminant validity, as the square root of the AVE for each construct exceeded its correlations with other constructs, consistent with the (Fornell and Larcker, 1981).

### 4.2 Structural equation modeling analysis

In summary, the fit of the structural equation model with the data ranged from acceptable to good. The overall validity of the model meets the fundamental standards of quantitative research. Based on TMIM, a structural equation model was specified and tested using path analysis in AMOS 26.0. The overall model fit indices are summarized in Table 4. The ratio of chi-square to degrees of freedom (χ^2^/*df* = 3.284) indicated an acceptable fit, considering the known sensitivity of the chi-square statistic to sample size (Marsh and Hocevar, 1985; Kline, 2023). The RMSEA value was 0.074, which was below the recommended cutoff of 0.08 and therefore suggested an acceptable fit (Browne, 1993). In addition, both the CFI (0.916) and IFI (0.916) exceeded 0.90, indicating satisfactory model fit (Hu and Bentler, 1999). Although the NFI value (0.884) was slightly below the 0.90 benchmark, it was close to the recommended threshold and was acceptable when considered together with the other fit indices (Marsh et al., 1988).

**Table 4:** Overall model fit indices.

Fit indices	Value	Criterion	Evaluation
Normed chi-square (χ^2^/*df*)	3.284	< 3 (good); 3-5 (acceptable)	Acceptable
Root Mean Square Error of Approximation (RMSEA)	0.074	< 0.08 (acceptable)	Acceptable
Comparative Fit Index (CFI)	0.916	>0.90 (acceptable)	Acceptable
Normed Fit Index (NFI)	0.884	>0.90 (acceptable)	Marginally acceptable
Incremental Fit Index (IFI)	0.916	>0.90 (acceptable)	Acceptable

The fit criteria refer to commonly accepted thresholds. Overall, the model demonstrated an acceptable fit to the data, although the NFI value was slightly below the recommended threshold.

Overall, the structural equation model showed an acceptable fit to the data. The path analysis results are presented in Table 5. The standardized path coefficient from uncertainty discrepancy to anxiety was β = 0.409 (SE = 0.137, *p* < 0.001), indicating a significant positive relationship and supporting H1. The path from anxiety to outcome expectancy was also significant and positive, β = 0.257 (SE = 0.026, *p* < 0.001), suggesting that higher anxiety was associated with higher outcome expectancy, thereby supporting H2.

**Table 5:** Path coefficients and significance levels of the structural equation model.

Path	Standardized path coefficient (β)	Standard error (SE)	Critical ratio (C.R.)	*p*-value	Significance	Hypothesis testing result
UD → AN (H1)	0.409	0.137	6.442	< 0.001	***	Supported
AN → OE (H2)	0.257	0.026	4.216	< 0.001	***	Supported
AN → EE (H3)	-0.157	0.029	-2.839	0.005	**	Supported
OE → EE (H4)	0.543	0.094	7.053	< 0.001	***	Supported
EE → IS (H5)	0.707	0.107	11.060	< 0.001	***	Supported
OE → IS (H6)	0.129	0.117	2.247	0.025	*	Supported

^*^Denotes *p* < 0.05, ^**^denotes *p* < 0.01, ^***^denotes *p* < 0.001; SE and C.R. are based on unstandardized estimates, whereas β represents standardized path coefficients; UD, uncertainty discrepancy; AN, anxiety; OE, outcome expectancy; EE, efficacy expectancy; IS, information seeking.

The standardized path coefficient from anxiety to efficacy expectancy was β = -0.157 (SE = 0.029, *p* = 0.005), indicating a significant negative effect of anxiety on efficacy expectancy, thus supporting H3. The standardized path coefficient from outcome expectancy to efficacy expectancy was β = 0.543 (SE = 0.094, *p* < 0.001), suggesting a strong positive predictive effect of outcome expectancy on efficacy expectancy, supporting H4. The standardized path coefficient from efficacy expectancy to information seeking was β = 0.707 (SE = 0.107, *p* < 0.001), revealing that efficacy expectancy is a key positive predictor of information seeking, supporting H5. The standardized path coefficient from outcome expectancy to information seeking was β = 0.129 (SE = 0.117, *p* = 0.025), indicating a significant positive predictive effect of outcome expectancy on information seeking, thus supporting H6.

In summary, all six core pathways proposed based on TMIM passed the significance tests. The path relationships in the model align closely with the theoretical framework.

To further examine the indirect mechanisms proposed by TMIM, bootstrap mediation analyses were conducted using SPSS PROCESS v4.1 (Hayes, 2017) with 5,000 bias-corrected bootstrap samples. The results are presented in Table 6.

**Table 6:** Supplementary PROCESS-based bootstrap analysis of completely standardized indirect effects based on composite scores.

Indirect pathway	Completely standardized indirect effect (β)	Bootstrapped (SE)	95% bias-corrected confidence interval (LLCI, ULCI)	Significance of indirect effect
AN → OE → IS	0.0747	0.0224	(0.0351, 0.1220)	Significant
AN → EE → IS	-0.0148	0.0345	(-0.0818, 0.0546)	Not significant
AN → OE → EE → IS	0.0457	0.0134	(0.0209, 0.0731)	Significant
UD → AN → OE → IS	0.0130	0.0081	(-0.0016, 0.0301)	Not significant
UD → AN → EE → IS	-0.0145	0.0136	(-0.0411, 0.0129)	Not significant
UD → AN → OE → EE → IS	0.0735	0.0166	(0.0434, 0.1087)	Significant

Bootstrap analyses were conducted using 5,000 bias-corrected bootstrap samples. Indirect effects were considered statistically significant when the 95% bias-corrected confidence interval did not include zero. UD, uncertainty discrepancy; AN, anxiety; OE, outcome expectancy; EE, efficacy expectancy; IS, information seeking. These coefficients were estimated using PROCESS based on observed scale composite scores and are not intended to be algebraically derived from the AMOS latent-variable standardized path coefficients reported in Table 5.

As shown in the Table 6, the indirect pathway from anxiety to information seeking through outcome expectancy was significant (β = 0.0747, 95% CI [0.0351, 0.1220]), whereas the indirect pathway through efficacy expectancy alone was not significant (β = −0.0148, 95% CI [-0.0818, 0.0546]). In addition, the sequential indirect pathway from anxiety to information seeking through outcome expectancy and efficacy expectancy was statistically significant (β = 0.0457, 95% CI [0.0209, 0.0731]).

Regarding the full TMIM chain, the indirect pathway from uncertainty discrepancy to information seeking through anxiety and outcome expectancy was not significant, nor was the pathway through anxiety and efficacy expectancy alone. However, the sequential indirect pathway from uncertainty discrepancy to information seeking through anxiety, outcome expectancy, and efficacy expectancy was significant (β = 0.0735, 95% CI [0.0434, 0.1087]). These findings provide additional support for the sequential cognitive appraisal process proposed by TMIM.

### 4.3 Validation of the adapted consumption-oriented new media literacy scale

Because the adapted consumption-oriented new media literacy (C-NML) scale was included only as a supplementary moderator variable in the PROCESS analyses rather than as part of the primary SEM measurement model, its psychometric properties were evaluated separately using confirmatory factor analysis (CFA) in AMOS 26.0. Consistent with the retained consumption-oriented dimensions of the original framework, a two-factor model consisting of functional consumption and critical consumption was specified to examine the factorial structure of the adapted scale. The psychometric evaluation results for the adapted C-NML scale are therefore reported separately from the primary SEM measurement model.

The CFA results indicated an overall acceptable model fit (χ^2^/*df* = 3.20, CFI = 0.909, TLI = 0.880, RMSEA = 0.073), with most fit indices meeting or approaching commonly recommended thresholds. In addition, all standardized factor loadings were statistically significant. Overall, these findings provide support for the factor validity and internal consistency of the adapted C-NML scale.

Given that the present study employed a contextually adapted version of the original new media literacy framework, the C-NML construct should be interpreted as a supplementary composite indicator reflecting students' consumption-oriented information access and evaluation competencies in the university-entry context, rather than as a complete representation of the original multidimensional new media literacy construct. Accordingly, the moderation findings involving C-NML should be interpreted with appropriate caution.

### 4.4 Testing for moderation effects

#### 4.4.1 Moderation effect of information overload

Moderation analyses were conducted using SPSS 27.0 and PROCESS v4.1 (Model 1) to test the moderating role of information overload (IO) in the relationships between anxiety (AN) and the evaluation-phase variables, following the regression-based procedures described in Hayes (2017). Bias-corrected bootstrap confidence intervals (5,000 resamples) were used for inference. All predictors were mean-centered, and gender was included as a covariate.

As shown in Table 7, after controlling for gender, the main effect of anxiety on outcome expectancy (OE) was significant (*B* = 0.139, SE = 0.025, *p* < 0.001), as was the main effect of information overload (*B* = −0.110, SE = 0.029, *p* < 0.001). Importantly, the interaction term between anxiety and information overload (AN × IO) also reached statistical significance (*B* = 0.060, SE = 0.021, *p* < 0.01), with Δ*R*^2^ = 0.018, F = 8.083, confirming a statistically significant interaction between anxiety and information overload.

**Table 7:** Results of moderation analysis for information overload on the anxiety and outcome expectancy relationship (controlling for gender).

Variable	Unstandardized coefficient (B)	Standard error (SE)	*t*-value	*p*-value	95% Confidence interval
Constant	4.068	0.082	49.521	<0.001	[3.907, 4.230]
Covariate: gender	-0.031	0.056	-0.543	0.587	[-0.142, 0.080]
Independent variable: anxiety (AN)	0.139	0.025	5.606	<0.001	[0.090, 0.188]
Moderator: information overload (IO)	-0.110	0.029	-3.775	<0.001	[-0.168, -0.053]
Interaction term: AN × IO	0.060	0.021	2.843	<0.01	[0.019, 0.102]

1. Gender was coded as 1 = female; 2 = male. 2. Sample size *n* = 417.

**Figure 3:**
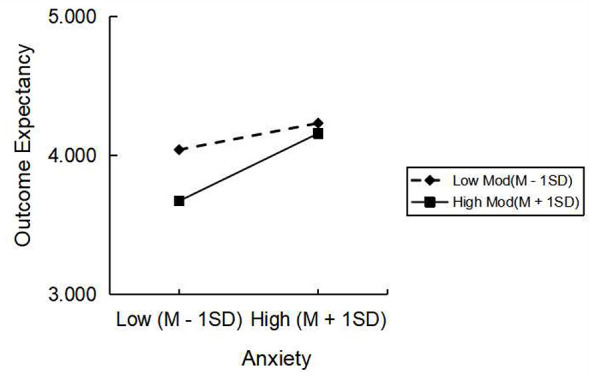
Moderation effect of information overload on the relationship between anxiety and outcome expectancy.

Simple slope analysis (see Table 8) further clarified the nature of this moderation. At a low level of information overload (M - 1SD = -1.004), anxiety was positively associated with outcome expectancy (*B* = 0.079, SE = 0.027, *p* < 0.005, 95% CI [0.025, 0.133]). At a high level of information overload (M + 1SD = 1.004), anxiety was also positively associated with outcome expectancy (*B* = 0.199, SE = 0.037, *p* < 0.001, 95% CI [0.126, 0.273]). As illustrated in Figure 3, the association between anxiety and outcome expectancy differed across low and high levels of information overload.

**Table 8:** Simple slope analysis results (effects of anxiety on the dependent variables at high/low levels of information overload).

Dependent variable	Information overload level (IO)	Unstandardized coefficient (B)	Standard error (SE)	*t*-value	*p*-value	95% Confidence interval
Outcome expectancy (OE)	Low (M-1SD, -1.004)	0.079	0.027	2.867	<0.005	[0.025, 0.133]
	High (M+1SD, 1.004)	0.199	0.037	5.367	<0.001	[0.126, 0.273]
Efficacy expectancy (EE)	N/A	N/A	N/A	N/A	N/A	N/A

N/A denotes not applicable. The anxiety × information overload interaction term was not significant for efficacy expectancy (*p* = 0.904); Therefore, the moderating effect was not established, and no simple slope analysis results are reported.

For efficacy expectancy (EE), the results presented in Table 9 indicated that neither the main effect of anxiety (*B* = −0.004, SE = 0.031, *p* = 0.884) nor the main effect of information overload (*B* = −0.017, SE = 0.036, *p* = 0.645) was statistically significant. In addition, the interaction term between anxiety and information overload was also non-significant (*B* = −0.003, SE = 0.026, *p* = 0.904), with Δ*R*^2^ = 0.000 and F = 0.014. Thus, information overload did not significantly moderate the relationship between anxiety and efficacy expectancy.

**Table 9:** Results of moderation analysis for information overload (IO) on the anxiety (AN) and efficacy expectancy (EE) relationship (controlling for gender).

Variable	Unstandardized coefficient (B)	Standard error (SE)	*t*-value	*p*-value	95% Confidence interval
Constant	3.532	0.102	34.673	< 0.001	[3.332, 3.732]
Covariate: gender	0.089	0.070	1.275	0.203	[-0.048, 0.227]
Independent variable: anxiety (AN)	-0.004	0.031	-0.146	0.884	[-0.065, 0.056]
Moderator: information overload (IO)	-0.017	0.036	-0.461	0.645	[-0.088, 0.055]
Interaction term: AN × IO	-0.003	0.026	-0.120	0.904	[-0.055, 0.048]

1. Gender was coded as 1 = female, 2 = male. 2. Sample size *n* = 417.

#### 4.4.2 Moderation effect of consumption-oriented new media literacy

Moderation analyses were conducted using SPSS 27.0 and PROCESS v4.1 (Model 1) to test the moderating effect of consumption-oriented new media literacy (C-NML) on the relationships between evaluation-phase variables and information seeking (IS), following the regression-based procedures described by Hayes (2017). Bias-corrected bootstrap confidence intervals (5,000 resamples) were used for inference. All predictors were mean-centered, and gender was included as a covariate. The sample comprised 417 participants.

As shown in Table 10, after controlling for gender, the main effect of outcome expectancy (OE) on information seeking was significant (*B* = 0.551, SE = 0.095, *p* < 0.001), whereas the main effect of consumption-oriented new media literacy was not significant (*B* = 0.188, SE = 0.118, *p* = 0.110). Importantly, the interaction term between outcome expectancy and consumption-oriented new media literacy (OE × C-NML) also reached statistical significance (*B* = −0.396, SE = 0.157, *p* < 0.05), with Δ*R*^2^ = 0.013, F = 6.384, confirming a statistically significant interaction between outcome expectancy and consumption-oriented new media literacy.

**Table 10:** Results of the moderating effect analysis of C-NML on the relationship between outcome expectancy (OE) and information seeking (IS) (controlling for gender).

Variable	Unstandardized coefficient (B)	Standard error (SE)	*t*-value	*p*-value	95% Confidence interval
Constant	5.566	0.137	40.703	<0.001	[5.297, 5.835]
Covariate: gender	-0.024	0.095	-0.252	0.801	[-0.210, 0.162]
Independent variable: outcome expectancy (OE)	0.551	0.095	5.820	<0.001	[0.365, 0.737]
Moderator: consumption-oriented new media literacy (C-NML)	0.188	0.118	1.600	0.110	[-0.043, 0.420]
Interaction term: OE × C-NML	-0.396	0.157	-2.527	<0.05	[-0.704, -0.088]

1. Gender was coded as 1 = female, 2 = male. 2. Sample size *n* = 417.

**Figure 4:**
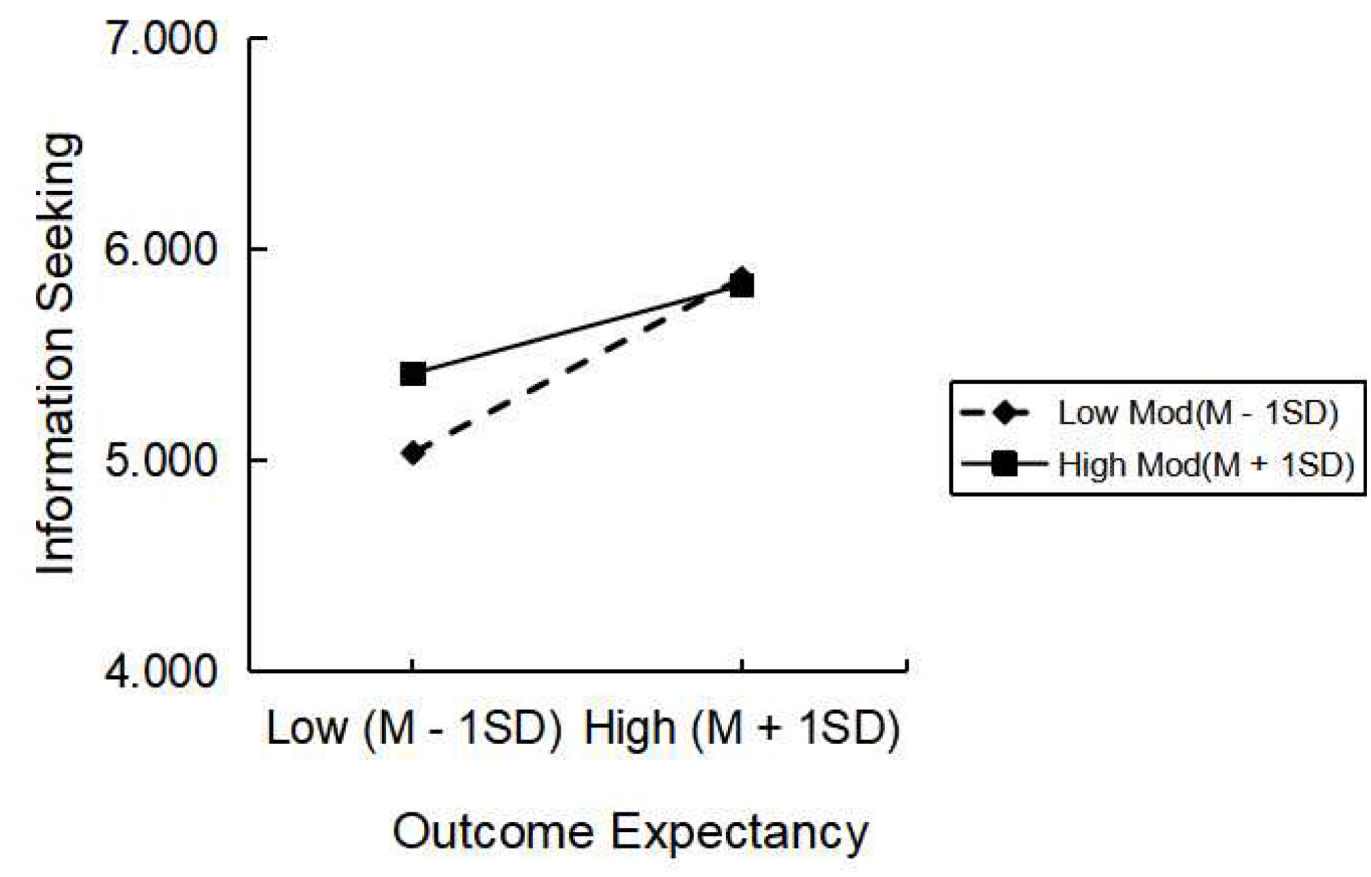
Moderation effect of consumption-oriented new media literacy on the relationship between outcome expectancy and information seeking.

Simple slope analysis (see Table 11) further clarified this interaction. At a low level of consumption-oriented new media literacy (M - 1SD = -0.457), outcome expectancy was positively associated with information seeking (*B* = 0.732, SE = 0.117, *p* < 0.001, 95% CI [0.501, 0.962]). At a high level of consumption-oriented new media literacy (M + 1SD = 0.457), outcome expectancy was also positively associated with information seeking (*B* = 0.370, SE = 0.120, *p* < 0.01, 95% CI [0.133, 0.606]). As illustrated in Figure 4, the association between outcome expectancy and information seeking differed across low and high levels of consumption-oriented new media literacy.

**Table 11:** Simple slope analysis results for the moderating effect of consumption-oriented new media literacy (C-NML) controlling for gender.

Dependent variable	Predictor	C-NML level	Simple slope (B)	SE	*t*-value	*p*-value	95% CI
Information seeking	Outcome expectancy	Low (M - 1SD, -0.457)	0.732	0.117	6.241	<0.001	[0.501, 0.962]
Information seeking	Outcome expectancy	High (M + 1SD, 0.457)	0.370	0.120	3.077	<0.01	[0.133, 0.606]
Information seeking	Efficacy expectancy	Low (M - 1SD, -0.457)	0.694	0.092	7.543	<0.001	[0.513, 0.875]
Information seeking	Efficacy expectancy	High (M + 1SD, 0.457)	0.973	0.066	14.686	<0.001	[0.843, 1.103]

**Figure 5:**
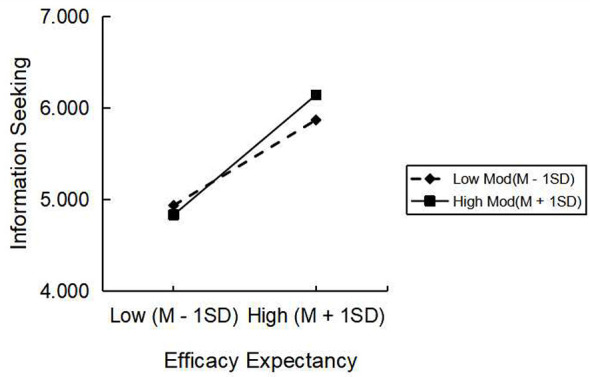
Moderation effect of consumption-oriented new media literacy on the relationship between efficacy expectancy and information seeking.

For the efficacy expectancy pathway, results in Table 12 indicate that, after controlling for gender, the main effect of efficacy expectancy on information seeking was significant (*B* = 0.833, SE = 0.065, *p* < 0.001), whereas the main effect of consumption-oriented new media literacy was not significant (*B* = 0.093, SE = 0.092, *p* = 0.316). The interaction term between efficacy expectancy and consumption-oriented new media literacy (EE × C-NML) was also statistically significant (*B* = 0.306, SE = 0.103, *p* < 0.01), with Δ*R*^2^ = 0.013, F = 8.780, indicating a moderating effect. Simple slope analysis (see Table 11) showed that at a low level of consumption-oriented new media literacy, efficacy expectancy was positively associated with information seeking (*B* = 0.694, SE = 0.092, *p* < 0.001, 95% CI [0.513, 0.875]). At a high level of consumption-oriented new media literacy, efficacy expectancy was also positively associated with information seeking (*B* = 0.973, SE = 0.066, *p* < 0.001, 95% CI [0.843, 1.103]). As illustrated in Figure 5, the association between efficacy expectancy and information seeking varied across different levels of consumption-oriented new media literacy.

**Table 12:** Results of the moderation analysis of C-NML on the relationship between efficacy expectancy and information seeking controlling for gender.

Variable	Unstandardized coefficient (B)	Standard error (SE)	*t*-value	*p*-value	95% Confidence interval
Constant	5.657	0.116	48.561	<0.001	[5.428, 5.886]
Covariate: gender	-0.156	0.080	-1.948	0.052	[-0.314, 0.001]
Independent variable: efficacy expectancy (EE)	0.833	0.065	12.855	<0.001	[0.706, 0.961]
Moderator: consumption-oriented new media literacy (C-NML)	0.093	0.092	1.003	0.316	[-0.089, 0.274]
Interaction term: EE × C-NML	0.306	0.103	2.963	<0.01	[0.103, 0.508]

1. Gender was coded as 1 = female, 2 = male. 2. Sample size *n* = 417.

## 5 Discussion

Guided by TMIM, this study examined the mechanisms and boundary conditions of information seeking among incoming first-year university students during the pre-enrollment period. The findings provide empirical support for the core pathways of TMIM in this population. Specifically, uncertainty discrepancy was positively associated with anxiety, which in turn was linked to information seeking through the mediating roles of outcome expectancy and efficacy expectancy. These results are generally consistent with prior TMIM-based research in health and family communication contexts, suggesting that the framework may also be useful for explaining information seeking during the transition to higher education.

One notable finding is that anxiety was positively associated with outcome expectancy in the context of university entry. This pattern differs from some earlier TMIM studies, which found negative associations between anxiety and outcome-related evaluations. In those contexts, anxious individuals may have expected greater costs or fewer benefits from seeking information. However, the present findings suggest that this relationship may work differently when information seeking is perceived as an actionable and preparatory coping strategy. For incoming first-year university students, pre-enrollment anxiety is often tied to concrete uncertainties about academic requirements, campus routines, accommodation, peer relationships, and future developmental opportunities. These uncertainties differ from many health-related uncertainties because they are relatively immediate, task-oriented, and potentially manageable through information acquisition. In this context, anxiety may increase students' awareness of information insufficiency and strengthen their belief that seeking university-related information can help them prepare for the transition and regain a sense of control.

Similar patterns have been observed in uncertainty-rich transitional contexts, where heightened anxiety can increase the perceived usefulness of information seeking as a way to regain control (Ju et al., 2022). This interpretation is also consistent with proactive coping and anticipatory socialization perspectives, which suggest that individuals often seek information before entering unfamiliar roles or environments to facilitate adaptation and reduce uncertainty (Fetherston, 2017). Likewise, during the transition to university, students may view information seeking as a proactive coping strategy that helps them prepare for upcoming academic, social, and institutional demands. Taken together, these findings suggest that the relationship between anxiety and outcome expectancy within TMIM may vary across informational and transitional contexts, rather than following a uniformly negative pattern.

With regard to the moderating effects, the findings reveal differentiated and context-dependent roles of information overload and consumption-oriented new media literacy within the TMIM process. Information overload moderated the relationship between anxiety and outcome expectancy, such that higher levels of overload strengthened the positive association between these two variables. Rather than uniformly impairing cognitive evaluation, information overload may heighten students' awareness of informational ambiguity and cognitive strain, thereby increasing their perceived need to clarify uncertain situations. Under such conditions, anxiety may serve as a cognitive cue that signals the need for action, which in turn enhances students' expectations regarding the usefulness of information seeking for uncertainty management and psychological relief. The non-significant moderating effect of information overload on the anxiety-efficacy expectancy relationship also deserves attention. This finding suggests that information overload may not shape all appraisal pathways in TMIM in the same way. One possible explanation is that information overload primarily changes students' judgments about the value and necessity of further information seeking, rather than their beliefs about their own ability to seek, communicate about, and cope with information. In the present study, efficacy expectancy mainly captured students' communication efficacy and coping efficacy. These efficacy beliefs may depend more on relatively stable personal resources, such as prior digital experience, communication confidence, help-seeking skills, and access to peers, senior students, or institutional support, than on the perceived amount of available information. Thus, while information overload strengthened the relationship between anxiety and outcome expectancy by increasing the perceived need for clarification, it did not significantly alter the relationship between anxiety and efficacy expectancy.

In contrast, the moderating role of consumption-oriented new media literacy exhibited a differentiated pattern across the two expectancy pathways. Consumption-oriented new media literacy strengthened the association between efficacy expectancy and information seeking, suggesting that students with higher media literacy are better able to translate perceived competence into actual information-seeking behavior. At the same time, consumption-oriented new media literacy weakened the association between outcome expectancy and information seeking, suggesting that students with higher media literacy may rely less on anticipated benefits alone when deciding whether to seek information. Instead, their behavior appears to be guided by a broader evaluation that integrates perceived ability, informational needs, and contextual considerations. In this sense, consumption-oriented new media literacy serves as a regulatory cognitive resource that recalibrates the basis of information seeking decisions, rather than uniformly amplifying or suppressing them. However, the present findings should be interpreted specifically as evidence concerning students' information consumption and evaluation competencies, rather than their broader participatory or content-production competencies in new media environments.

Compared with existing research, this study extends the application of TMIM to the educational transition context, complementing prior work by Feng et al. (2025). By focusing specifically on the period surrounding university matriculation, this study highlights the distinct informational ecology that incoming first-year university students face. Unlike health-related contexts where information is often acute and threat-driven, the informational environment associated with university entry is characterized by sustained uncertainty and ongoing decision demands. This context helps explain the observed expectancy patterns during the university transition. Moreover, by incorporating information overload and consumption-oriented new media literacy as moderating variables, the present study provides a more nuanced account of how emotional, cognitive, and environmental factors jointly shape information-seeking behavior.

From a practical perspective, the findings have implications for the design of pre-enrollment support systems in Chinese universities. First, universities should not only increase the amount of information provided to incoming students, but also improve the structure, clarity, and accessibility of that information. In the Chinese higher education context, incoming students often rely heavily on fragmented information obtained from platforms such as Xiaohongshu, WeChat, online forums, and short-video applications. Since information overload strengthened the relationship between anxiety and outcome expectancy, institutions should provide structured and prioritized information through centralized pre-enrollment platforms. Key information regarding registration, accommodation, course selection, financial aid, counseling services, and campus resources should be organized according to different stages of the transition process rather than being scattered across multiple platforms.

Second, orientation programs and student counseling services should recognize transition-related anxiety as a signal of unmet information needs. The positive association between anxiety and outcome expectancy suggests that anxious students may perceive information seeking as a useful preparation strategy. Universities could therefore provide guided information-seeking tasks, online question-and-answer sessions, peer mentoring programs, and brief psycho educational materials before enrollment to help students turn anxiety into constructive preparation.

Third, academic libraries could play a more active role in supporting first-year students'transition adjustment. Because consumption-oriented new media literacy influenced the translation of cognitive appraisals into information seeking, libraries could offer brief information literacy interventions before or during orientation. Such interventions may help students identify official information sources, critically evaluate social media content, distinguish personal experiences from institutional policies, and use online information tools more effectively when seeking university-related information.

Despite these contributions, several limitations should be acknowledged. First, although the snowball sampling strategy allowed us to reach respondents from a wide range of regions, the reliance on social media-based recruitment may have introduced selection bias. Because snowball sampling tends to propagate through existing social networks, individuals who are more digitally active and more embedded in online information environments are more likely to be recruited. As a result, students with limited access to digital resources or lower levels of digital literacy may have been underrepresented. This concern may be particularly relevant for students from rural or socioeconomically disadvantaged backgrounds who may have relatively limited digital access or lower levels of online engagement. Consequently, the representativeness of the sample and the external validity of the findings may be constrained, particularly with regard to populations who are less engaged in online information-seeking activities. Therefore, caution is warranted when generalizing the findings to the broader population of incoming first-year university students, especially those with lower levels of digital engagement.

Second, the cross-sectional design limits the ability to establish causal relationships among the variables. Because all variables were measured at a single time point before university entry, the structural paths should be interpreted as theoretically specified associations rather than causal effects. In addition, the present study cannot determine whether pre-enrollment information seeking contributes to subsequent post-enrollment outcomes, such as academic adjustment, social integration, or psychological adaptation. Since this study focused specifically on anticipatory information management before matriculation, the findings may not fully generalize to information behaviors that emerge after students formally enter university. Future longitudinal or experimental research could further examine how uncertainty discrepancy, anxiety, expectancy evaluations, and information seeking evolve across different stages of university adjustment.

Third, although Harman's single-factor test suggested that common method bias was not a serious concern in this study, all variables were measured using self-reported questionnaires collected at a single time point. Therefore, future studies could collect data from multiple sources, adopt time-lagged designs, or combine survey data with behavioral trace data to further reduce the potential influence of common method bias.

Fourth, consumption-oriented new media literacy was operationalized through its consumption-oriented dimensions, namely functional consumption and critical consumption. Although this operationalization was theoretically consistent with the study's focus on information seeking, it did not cover the full four dimensional structure of new media literacy, particularly the functional and critical prosuming dimensions. Accordingly, the findings should not be generalized to students' broader participatory or content-production competencies in new media environments. Future research could incorporate the complete four-dimensional framework and further examine whether prosuming-related competencies play distinct roles in post-enrollment peer communication, online community participation, or university-related content sharing.

Finally, the AVE values for outcome expectancy and efficacy expectancy were slightly below the conventional 0.50 threshold. Although their CR values and factor loadings were acceptable, findings involving these two constructs should be interpreted with caution.

Building on these findings, future research could pursue several directions. Longitudinal designs tracking students from admission through the first year of study would allow for a more detailed examination of the dynamic adjustment processes underlying information seeking. Comparative analyses could further explore subgroup differences, such as variations between first-generation college students and their peers or between students from urban and rural backgrounds. Theoretically, incorporating variables such as perceived information credibility and social support networks may contribute to a more comprehensive explanatory model. Methodologically, integrating qualitative approaches, including interviews and diary studies, could offer deeper insight into the contextualized cognitive and emotional processes that shape incoming first-year university students' information-related decisions.

## 6 Conclusion

This study systematically examined the psychological mechanisms and contextual boundary conditions underlying incoming first-year university students' information seeking by integrating TMIM with features of the contemporary digital information environment. The findings indicate that uncertainty related to university life was associated with heightened anxiety, which was in turn linked to information seeking through the evaluative pathways of outcome expectancy and efficacy expectancy.

In addition, the results show that contextual and individual factors played important moderating roles in this process. Information overload moderated the relationship between anxiety and outcome expectancy, suggesting that students' evaluations of the usefulness of information seeking were contingent on the surrounding informational environment. Consumption-oriented new media literacy also exhibited differentiated moderating patterns across the TMIM pathways: it shaped how efficacy expectancy was translated into information seeking and influenced the extent to which outcome expectancy informed information seeking decisions. Together, these findings highlight the conditional and context-sensitive nature of information seeking in digitally saturated settings.

This study makes two primary theoretical contributions. First, it extends TMIM to the university entry transition, providing evidence that the model can be applied beyond its traditional health and family communication contexts. Second, by incorporating information overload and consumption-oriented new media literacy as moderating variables, the study offers a more nuanced understanding of how emotional states, cognitive evaluations, and digital competencies jointly shape information management processes. Future research could extend this line of inquiry in several directions. Cross-cultural comparative studies may reveal how differences in educational systems and sociocultural contexts shape information seeking during transitional periods.

Future studies could also further examine the differentiated roles of communication efficacy, coping efficacy, and target efficacy within the TMIM framework, especially whether information overload affects these efficacy dimensions differently. Longitudinal designs would help clarify how information seeking, academic adjustment, and psychological adaptation dynamically interact over time during the university transition.

## Data Availability

The original contributions presented in the study are included in the article/Supplementary material, further inquiries can be directed to the corresponding author.

## Footnotes

1
https://hudong.moe.gov.cn/jyb_sjzl/sjzl_fztjgb/202506/t20250611_1193760.html

2
https://www.cnnic.net.cn/n4/2025/0721/c88-11328.html
